# Adaptation and psychometric testing of the hoarding rating scale (HRS): a self-administered screening scale for epidemiological study in Chinese population

**DOI:** 10.1186/s12888-020-02539-7

**Published:** 2020-04-14

**Authors:** Tai Wa Liu, Simon Ching Lam, Man Hon Chung, Ken Hok Man Ho

**Affiliations:** 1 School of Nursing and Health Studies, The Open University of Hong Kong, Ho Man Tin, Hong Kong SAR, China; 2 Department of Rehabilitation Sciences, The Hong Kong Polytechnic University, Hung Hom, Hong Kong SAR, China; 3 School of Nursing, The Hong Kong Polytechnic University, Hung Hom, Hong Kong SAR, China; 4 School of Nursing, Tung Wah College, Ho Man Tin, Hong Kong SAR, China

**Keywords:** Hoarding behaviors, Obsessive-compulsive disorder, Adaptation, Validation, Factor analysis, Psychometric properties

## Abstract

**Background:**

Hoarding disorder is a chronic and debilitating illness associated with restrictions on activities of daily living, compromised social and occupational functioning, and adverse health outcomes. However, researchers lack a brief and self-administered screening measurement to assess compulsive hoarding in the Chinese speaking population. This study aimed to adapt and validate the Hoarding Rating Scale-Interview (HRS-I) to as a tool for screening compulsive hoarding behavior in Chinese population.

**Methods:**

This study comprised two phases. During Phase 1, the English-language HRS-I was translated into Chinese (CHRS) (comprehensible for most Chinese speaking population, e.g., Cantonese & Mandarin) and subjected to an equivalence check. In Phase 2, the CHRS was validated by examining internal consistency, stability, and construct validity. Different samples were used appropriately to verify the items and reflect the psychometric properties.

**Results:**

In Phase 1, the CHRS yielded satisfactory content (S-CVI = 0.93) and face validity ratings (comprehensibility = 100%, *N* = 20 participants of general public with age 18–72) and the English and Chinese versions were found to be equivalent (ICC = 0.887; *N* = 60 university staff and students). Phase 2 revealed satisfactory levels of internal consistency (Cronbach’s α = 0.86; corrected item-total correlation = 0.60–0.74; *N* = 820 participants of general public), 2-week test-retest reliability (ICC = 0.78; *N* = 60 university students), and construct validity (one-factor CFA solution matched with the hypothesized model, χ^2^/d.f. = 2.26, RMSEA = 0.049, CFI = 0.99, IFI = 0.99, NFI = 0.99; *n* = 520 participants of general public).

**Conclusions:**

This study provides sufficient evidence of the reliability and validity of the CHRS for compulsive hoarding behavior screening in the Chinese population through self-administered method.

## Background

Compulsive hoarding is defined as the compulsive acquisition of objects and difficulties with discarding clutter to the extent that personal living space is severely affected [41]. Individuals exhibiting compulsive hoarding demonstrated increases risk of falls and fire hazards, head injuries, arthritic conditions, relative to healthy individuals [2]. Internationally, 2–5% of general public suffer from compulsive hoarding [8, 33] and the lifetime prevalence rates is as high as 5% [37]. In Chinese population, apart from a small scale study on 139 patients with obsessive-compulsive disorder indicating a prevalence of 8.6% hoarding symptom of them [30], there is a limited number of epidemiological research reporting the phenomenon of compulsive hoarding in general public. Given the onset of compulsive hoarding symptoms may occur early (i.e., childhood) [21, 41] and progress throughout life [21], having a screening tool for compulsive hoarding is crucial for early detection. Historically, compulsive hoarding was considered as a subtype of obsessive-compulsive disorder (OCD) [32] and was assessed in previous studies [15, 16] using the Yale-Brown Obsessive Compulsive Scale (Y-BOCS [20];). However, the use of an OCD instrument to assess compulsive hoarding is subject to two main methodological limitations. First, assessing hoarding under the context of OCD would mean that essential features of hoarding disorders, such as the severity of cluttering and associated functional impairments, would not be considered [17]. Second, people with hoarding disorders might not regard their hoarding behavior as “obsessive” or “compulsive” [17]. Accordingly, the validity of these two Y-BOCS items to assess compulsive hoarding could be jeopardized.

The 5-item Hoarding Rating Scale-Interview (HRS-I) [41] is trustworthy to assess and screen hoarding in accordance with the diagnostic criteria of the Diagnostic and Statistical Manual of Mental Disorders (DSM-5 [1];). The HRS-I assesses several domains of compulsive hoarding, including the level of cluttering, excessive acquisition, difficulties of discarding, associated distress and functional impairment [41]. The original HRS-I was scored on a nine-point scale (0 = none, 8 = extreme) and demonstrated a Cronbach’s alpha of 0.87 to 0.97 for internal consistency and excellent test-retest reliability (r = 0.96; intraclass coefficients = 0.81–0.85) in a validation study of 87 [42] to 136 [41] subjects with or without compulsive hoarding or OCD. The HRS-I also yielded good known-group validity in the discrimination of hoarding and non-hoarding participants with or without OCD [41]. Furthermore, the HRS-I scores exhibited significant strong correlations with other validated hoarding measures (r = 0.72–0.89, *p* < 0.001), including the Savings Inventory Revised, Clutter Image Rating [17] and Obsessive-Compulsive Inventory-Revised [14], in an analysis of convergent validity.

Given the increasing prevalence of hoarding and the need to early diagnoses hoarding in Chinese population, clinicians and researchers would benefit from having a self-administered Chinese HRS (CHRS) to assess and screen people with compulsive hoarding. Therefore, this study had the following objectives: (1) to translate the HRS-I from English into Chinese; (2) to psychometrically test the Chinese version of the HRS (CHRS), including content validity, internal consistency, test-retest reliability and structural validity in a general population of Hong Kong.

## Methods

**Fig. 1:**
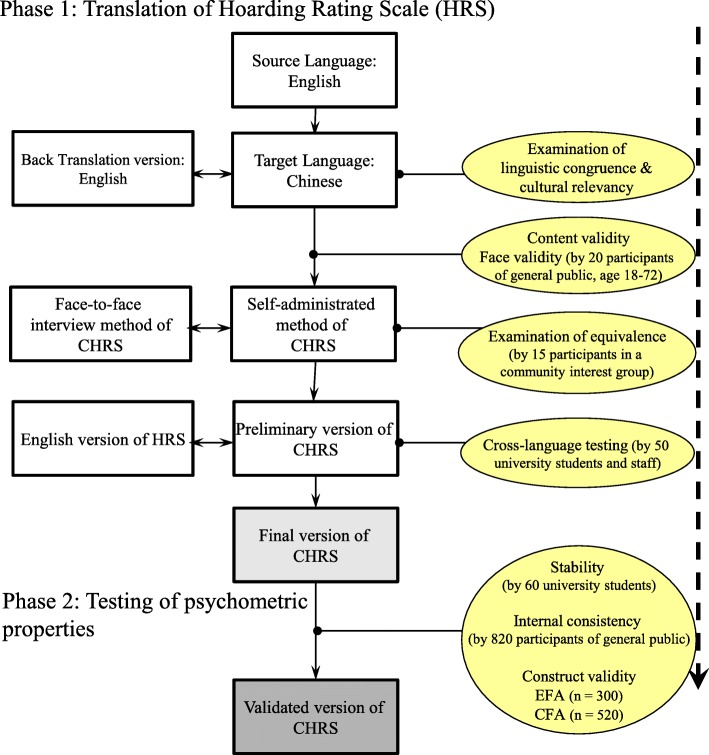
A logistic flow chart of the translation and validation methodology

This research used a cross-sectional methodological design in two phases. Phase 1 aimed: (i) to translate the English-language version of the HRS-I into the traditional Chinese language (the most common and comprehensible language used by Chinese speaking people worldwide) [12], (ii) to examine the relevancy and comprehensibility of this version, and (iii) to evaluate the translation equivalence. The second phase examined the psychometric properties of the traditional Chinese-language version (hereinafter referred to as CHRS). Figure 1 illustrates the entire process of adaptation and validation.

### Phase 1: translation of hoarding rating scale

The translation process was based on the principles of Brislin’s model of forward and backward translation [4]. Two independent translators (bilingual, PhD in Nursing and Social Science, experience in mental health) translated the HRS-I [41] from its source language (SL; English) to the target language (TL; Traditional Chinese). The TL version was then reviewed by a Chinese monolingual reviewer to identify ambiguous and unclear wordings, which were modified by the research team. A bilingual expert (holding a PhD in translation) compared the back-translated version (BT; English), which was translated by an academic staff in mental health professor, with the SL version to evaluate the cultural relevancy as well as linguistic congruence. Subsequently, the research team reviewed and discussed any incongruence in translation or difficulties encountered by the expert. The abovementioned process was repeated until there was no loss of any essential and stem meaning of each statement between the SL and BT, which indicated a maximum agreement.

#### Content and face validation

Six experts in healthcare and social science disciplines (including a psychiatrist, nurses working in mental health, and academic staff in psychology and sociology) were invited to examine the relevancy of CHRS and establish content validity using a four-point Likert scale (from 1 = not relevant to 4 = highly relevant) [34]. Panel members were identified by their publications and expertise in the university profiles as well as hospital services, hence, they were of competent knowledge experience in the subject matter. Expert panel members who gave ratings of ≤3 were required to provide feedback. The content validity index (CVI), “which indicates the proportion of responses that agree with the relevancy of the scale, was computed based on the mean ratings given by experts that gave ratings of 3 or 4.” ([26], p. 761). The scale- (S-CVI) and item-level CVIs (I-CVI) were of 0.80 or above considered satisfactory [27, 34].

Face validity of the CHRS was examined to assess the comprehensibility of each items by the general public, which aimed to ensure the applicability of a self-report method. A purposive sample of 8 to 20, including an appropriate good mix of demographics (e.g., men and women, young and old adults, highly and less educated), were recruited for the face validation process because the literature indicated that this sample size can sufficiently detect ambiguous items [28, 39]. These participants were invited to comment each item regarding comprehensibility (i.e., rated on yes/no) and to rephrase them by their own words to check its interpretability (the researcher rated the participants’ answers on a 4-point Likert scale regarding interpretability, 1 = fully correct to 4 = completely wrong) [24, 25]. The evaluation of comprehensibility was conventionally applied by other studies for face validation [35], while the latter method (some school of thoughts regarded it as “cognitive debriefing/interview”) was deemed to be relatively more sensitive and specific for identifying problematic items [24, 25]. If necessary, the participants would suggest some wordings and sentence styles for the items to improve interpretability. After that, a preliminary version of the CHRS was established.

To establish equivalence between the interview-based (HRS-I) and self-administered (CHRS) methods, a convenience sample of 15 participants recruited at a community interest group was initially invited to respond to the CHRS, followed by a face-to-face interview with a psychiatric nurse 2 weeks later. The equivalence between the two methods was computed using an intraclass correlation coefficient (model 3) (ICC ≥ 0.75 indicated a satisfactory equivalence) [35].

#### Cross-language testing

Cross-language testing is considered the most stringent method for examining translation equivalence [23, 27]. The translation equivalence of the HRS-I and CHRS was examined in a convenience sample of 60 teaching staff and undergraduate students recruited from a university in Hong Kong. These participants were selected because of their capability to understand the items of both the HRS-I and CHRS. The participants first responded to the HRS-I, followed by the CHRS 2 weeks later. All participants were indexed using an individual anonymized self-generated code for internal matching purposes. The scores compared between the HRS-I and CHRS was analyzed by the ICC (≥ 0.75, indicating satisfactory translation equivalence) [27, 35].

### Phase 2: Psychometric testing of the Chinese version of the hoarding rating scale

A correlational and cross-sectional design was adopted for the psychometric testing of CHRS, which evaluated the internal consistency, stability, and construct validity. In order to provide independent evaluation of psychometric properties and avoid psychological carry-over effects, samples used in phase 1 were excluded. The participants were newly recruited from the general public in three major districts in Hong Kong—Hong Kong Island, New Territories, and Kowloon—which included a good mix of participants with different socio-demographics [26]. A research nurse invited pedestrians to respond self-administrated questionnaires, including demographics and the CHRS by the use of paper-and-pencil method. Sample size was about 800; of these, 300 participants were randomly selected for exploratory factor analysis (EFA) and the remaining 500 were included in a confirmatory factor analysis (CFA). The estimated sample size was considered good for CFA [27, 40] and sufficient for the psychometric evaluation described below.

#### Reliability of the Chinese version of the hoarding rating scale

The internal consistency of the CHRS was computed using conventional “Cronbach’s α statistics (where α ≥ 0.70 indicates a satisfactory result) and the corrected item-total correlation coefficient (where r ≥ 0.30 indicates a homogenous item)” ([26], p.763). Stability was examined by test-retest reliability over a 2-week period. Because of the principle of anonymity, the samples recruited from the general public did not contain any contact information, which made impossible for retest. A convenience sample of 62 undergraduate students was selected to answer the questionnaire twice (T1 as well as T2, 2 weeks later) [26]. The anonymous T1 and T2 responses collected from each student were matched using self-generated codes (i.e., combinations of mobile and student identity numbers) as described in a previous study [28]. A formula (expected ICC = 0.80, and 95% confidence interval [CI] for ICC = 0.20) with consideration of attrition rate of 20% was adopted to suggest a sample size of 62 [19]. The ICC (model 3) (≥ 0.75 indicates a satisfactory stability) was used to compare the T1 and T2 scores [27, 35].

#### Construct validity of the Chinese version of the hoarding rating scale

Construct validation was evaluated by checking the factorial structure of the CHRS. As the developers of the HRS-I did not present the factor model, an EFA was initially used to explore the factorial structure of the CHRS. A scree plot was generated using a maximum likelihood analysis (for normally distributed data) or principal axis factoring (for non-normally distributed data) to illustrate the number of factors to be extracted [10]. We used the Promax rotation method for oblique rotation with Kaiser normalization to produce the best factor solution. Data factorability was evaluated using Bartlett’s test of sphericity (*p* < 0.001) and the Kaiser–Myer–Olkin (KMO) index (> 0.6) [40]. The factor loading of each item to the respective latent factor should exceed 0.40 [13]. Data from 300 randomly selected participants in the data pool were used to compute the EFA.

The degree of fitness of the data in a hypothesized model (i.e., the model identified by EFA) was then re-evaluated by CFA. Goodness-of-fit measures, namely the chi-square/degree of freedom ratio (χ^2^/d.f.), root mean square error of approximation (RMSEA), comparative fit index (CFI), incremental fit index (IFI), and normed fit index (NFI), were used to evaluate the model fit. These measures yielded goodness-of-fit indices of χ^2^/d.f. < 5.00 [9, 22], RMSEA < 0.08, CFI, IFI, and NFI > 0.90 and [6, 9, 27]. Data from the remaining 500 participants were used to generate the CFA model.

### Instrument

The CHRS derived through Phases 1 and 2 of this study was used to measure CHB. The original HRS-I comprised five items: severity of cluttering, difficulty discarding, excessive acquisition, distress and functional impairment associated with hoarding [41]. Each item was rated on a 9-point scale (0 = none/no problem, 2 = mild/occasionally, 4 = moderate/regularly, 6 = severe/frequently, 8 = extreme/very often, depending on the question). The total CHRS score was computed by summing all items, with a possible range of 0–40. A higher score indicated more severe CHB. The optimal cut-off score of 14 was determined to have a both a sensitivity and specificity of 0.97 for indicating CHB [41] and the latest analysis indicated a cut-off score of 11 that showed excellent sensitivity (1.00) and specificity (1.00) for distinguishing the hoarding disorder group and healthy control group [42].

### Data analysis

The Statistical Product and Service Solutions software, version 22.0 for Windows (IBM SPSS Inc.), was used for the analysis. Descriptive statistics (e.g., means, standard deviations and percentages) and inferential statistics (e.g., Cronbach’s α, ICC, t-test, statistics used in the EFA) were used as described above. AMOS version 7.0 (IBM SPSS Inc.) was used for the CFA. A *p* value of < 0.05 was used to indicate statistical significance.

### Ethics

The research team reproduced and translated the HRS-I with permission from corresponding copyright holders. Appropriate methods were used to obtain consent from the participants, including implied verbal consent from participants who were recruited among the general public in railway stations and written consent from undergraduate students and university staff recruited on campus. Ethical approval was obtained from the ethical committee of a local university and the collaborative organization regarding the research purpose, methods of data collection (i.e., both verbal and written consent) and so on.

## Results

### Phase 1 results

The HRS-I was translated from English to traditional Chinese. The Chinese monolingual reviewer reported that there was no ambiguity in the TL version. The relevancy and linguistic congruence of both the SL and BT was further confirmed by the linguistic expert, who also commented that the basic meanings of the items were maintained in the translation.

The HRS-I had adopted an interview method involving probing with follow-up questions, and the raters made an independent rating of severity [41]. For content validation, therefore, the experts strongly suggested that the CHRS, as a self-administered scale, should include several items to recap the current condition of stocking (not adding items to this construct, and instead interpreting these items as probing or warm-up questions). The research team accepted this constructive suggestion and added four stocking questions prior to developing the CHRS. These questions asked the respondents to review the numbers of shoes, bags, t-shirts and any other items stored in their houses in large amounts. After this addition, the six healthcare and social science experts commented that the CHRS was satisfactorily relevant, as reflected by a S-CVI of 93.3% and I-CVI of 80.0–100%. For face validation, twenty participants (60% female) aged 18–72 years and with education levels ranging from primary school to a master’s degree commented that the CHRS items were comprehensible, yielding a comprehensibility and interpretability rating of 100%. In order to test the equivalence between the interview-based (HRS-I) and self-administrated (CHRS) methods, a sample of 15 participants was retested twice during 2 weeks interval (mean self-administered CHRS score = 14.47, SD = 6.32; mean HRS-I interview score = 13.60, SD = 6.25), the ICC was 0.852 (95% CI: 0.616–0.948, *p* < 0.001). To test the translation adequacy, a total of 60 undergraduate students and university staff were invited to respond to both the English and traditional Chinese versions of the HRS-I. Fifty participants (response rate = 83.3%) responded to both versions with duration of 2 weeks, and the ICC was 0.887 (95% CI: 0.809–0.934, *p* < 0.001).

### Phase 2 results

Recruitment from the general public yielded 921 completed questionnaires. Of these, 101 responses were discarded due to the incompleteness of CHRS items (*n* = 24) and acquiescence response (*n* = 77). Finally, 820 responses were included in the analysis (42.0% male, 59.0% single, 67.2% with tertiary education or higher and 62.5% with a monthly income less than USD 2564) (refer to Table 1 for details).

**Table 1:** Demographic characteristics of participants (*N* = 820)

Demographic Characteristics	Frequency	Percentage
Age range, years		
18–29	368	44.9
30–39	179	21.8
40–49	113	13.8
50–59	123	15.0
≥ 60	36	4.4
Missing	1	0.1
Gender		
Male	344	42.0
Female	470	57.3
Missing	6	0.7
Marital status		
Single	484	59.0
Married/co-habit	335	40.9
Missing	1	0.1
Education background		
Primary school or below	56	6.8
Secondary school	212	25.9
Tertiary school or above	551	67.2
Missing	1	0.1
Income range^a^, USD (HKD)		
< 1282 (< 10,000)	175	21.4
1283–2564 (10,001–20,000)	337	41.1
2565–5128 (20,001–40,000)	211	25.7
5129–7692 (40,001–60,000)	72	8.8
≥ 7693 (≥60,001)	24	2.9
Missing	1	0.1

^a^ USD to HKD exchange rate is generally based on a ratio of 1 to 7.8

The Cronbach’s alpha of the CHRS was 0.86, with corrected item-total correlation coefficients of 0.60–0.74. All coefficients indicate that the scale has satisfactory internal consistency. Subsequently, with two attrition cases (3.2%) because of incompletion of questionnaire twice, the test-retest reliability of CHRS was computed based on the data of 60 undergraduate students. The ICC of 0.78 (95% CI: 0.63–0.88) indicated satisfactory stability.

Prior to the factor analyses, the univariate and multivariate normality of item responses from 820 samples were checked. Although univariate normality was supported (i.e., skewness value = − 0.017 to 0.481; kurtosis value = − 0.798 to − 0.478), multivariate normality was slightly violated (multivariate kurtosis value = 6.275, critical ratio = 10.738). To explore the previously unexamined structure of the CHRS, an EFA was conducted using 300 randomly selected datasets from the abovementioned 820 samples. The data factorability was satisfactory according to the KMO (0.809) and significant Bartlett’s test of Sphericity (χ^2^ = 777.25, *p* < 0.001). With a non-normally distributed data, a principal axis factoring was used. The EFA indicated that the CHRS had a single factor structure, and 65.84% of the total variance was explained. The item loadings on this construct ranged from 0.69 to 0.85.

**Fig. 2:**
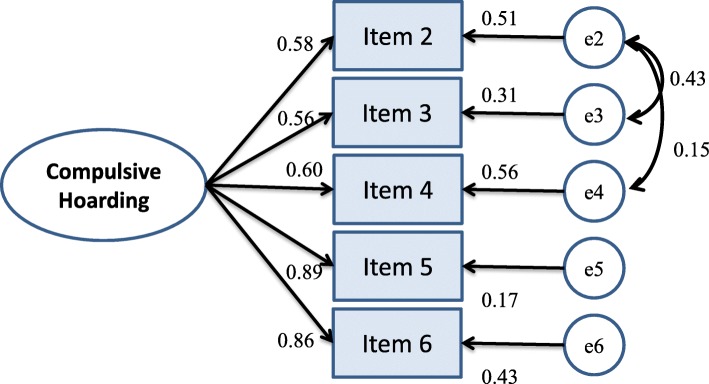
Confirmatory factor analysis model of the Chinese version of the Hoarding Rating Scale (CHRS). Remark: Item 1 is for recapping the condition of stocking, which serves as probing question to facilitate the participants’ responses on item 2 to 6

The 520 remaining datasets were included in a CFA, which indicated that all paths were significantly loaded to a single factor construct (range of loadings: 0.58–0.90). The preliminary goodness-of-fit indices revealed a marginal fit of the data model (χ^2^/d.f. = 23.66, RMSEA = 0.209, CFI = 0.90, IFI = 0.90, NFI = 0.90). A Bollen-Stine bootstrapping (with 2000 bootstraps) was used to provide a better adjustment of the χ^2^ and *p*-value for the non-normality of the estimation [3]. The results also rejected the current model fit. With reference to the covariance modification indices, two pairs of error terms with the largest indices (first covaried errors of items: 1 and 2, and second covaried errors of items: 1 and 3) could be covaried to improve the model fit [18, 27]. The corrected model yielded satisfactory goodness-of-fit indices (χ^2^/d.f. = 2.26, RMSEA = 0.049, CFI = 0.99, IFI = 0.99, NFI = 0.99) in this single factor model (refer to Fig. 2 for the CFA model). The results of Bollen-Stine bootstrapping also accepted this model fit (rejected null hypothesis with *p* = 0.068) with adjusted χ^2^ of 4.04 (i.e., bootstrap maximum likelihood estimation of χ^2^/d.f. = 1.35). Table 2 summarizes the psychometric properties of the CHRS. Additional file 1 includes the final version of the CHRS and Additional file 2 is the self-administered English version of HRS.

**Table 2:** Summary of the psychometric properties of the Chinese version of the Hoarding Rating Scale-Interview (CHRS)

	Methods	Statistic methods	Results
Reliability			
1. Internal consistency	Cronbach’s method Corrected item-total correlation	Cronbach’s alpha statistic Pearson moment-product correlation coefficient	Alpha of scale = 0.86 Corrected item-total correlation = 0.60–0.74
2. Stability	2-week test-retest reliability ^a^	Intraclass correlation coefficient (ICC)	*r* = 0.78, *p* < 0.001 95% CI: 0.63–0.88
Validity			
1. Face validity	Review by target population ^b^	Frequency & percentage	100% comprehensibility and interpretability
2. Content validity	Review by expert panel	Content validity index (CVI)	I-CVI = 0.80–1.00, S-CVI = 0.93
3. Construct validity 1.	Factor analysis	Exploratory factor analysis ^c^	KMO = 0.809 Bartlett’s Test of Sphericity: χ^2^ = 777.25, *p* < 0.001 Total variance explained = 65.84% Item loadings = 0.69–0.85
		Confirmatory factor analysis ^d^	χ^2^/d.f. = 2.26, CFI = 0.99, NFI = 0.99, IFI = 0.99, RMSEA = 0.049 (First-order one-factor CFA model)

Remarks:

*CI* Confidence interval

*I-CVI* Item-level content validity index

*S-CVI* Scale-level content validity index on average

^a^ The result was calculated based on 60 undergraduate students

^b^ The result was calculated based on 20 participants of general public (aged 18–72 years)

^c^ The result was calculated based on 300 randomly selected samples from among 820 samples

^d^ The result was calculated based on the remaining 520 samples not used to compute the EFA

## Discussion

This study was the first to translate the HRS-I into the traditional Chinese language using a recommended standard procedure. The traditional Chinese is the most common comprehensible language for Chinese people in mainland China, Taiwan, Hong Kong and even any countries in the world [12]. The items of HRS-I was developed in accordance with the diagnostic criteria of compulsive hoarding in DSM-5 [41]. We believed that the cultural variation for determining the compulsive hoarding is not a concern because, as indicated in the literature, HRS have been translated into different languages and used in different ethnic groups without any major change on items [11, 29, 43, 44]. Although the samples used for this study are Hong Kong people, the application of CHRS in broader Chinese population should be still appropriate.

Up to our knowledge, it is the first study to establish the equivalence between English interview version and Chinese self-administered version, which greatly facilitated a large-scale population-based research on this area. Our findings contribute to the development of a CHB instrument by expanding the reliability and validity of the original English-language HRS-I for the assessment of CHB in a general population. Our CFA analysis proposed a five-item, one-factor structure for the CHRS, which is consistent with the DSM-5 and the other hoarding measurement [1, 7].

The goal of an internal consistency assessment is to evaluate the item-level and overall consistency of an instrument intended to measure the same traits of the construct of interest. A satisfactory Cronbach’s alpha value of a measurement often represents the homogeneity of items, but up to a point of 0.90 or above, it might suggest redundancy of items [38]. For the high Cronbach’s alpha value (0.97) reported in the original English version of the HRS-I [41], one possible explanation is that more than half of the study participants (*n* = 73, 53%) were people being identified with compulsive hoarding preceded to the study. As this cohort of participants already possessed the traits of compulsive hoarding, it is plausible that a high than the desired Cronbach’s alpha value was obtained and reflected by the HRS-I. This is because the development of HRS-I is consistent with the diagnostic criteria listed in the DSM-5 regarding compulsive hoarding [1]. Thus, when being applied in general population, our results revealed that the CHRS exhibited satisfactory internal consistency with a Cronbach’s alpha of 0.86 [35]. Furthermore, the CHRS also yielded a satisfactory corrected item-total correlations (*r* = 0.60–0.74), which might suggest an optimal level of internal consistency with no redundancy (α > 0.90) or heterogeneity (α < 0.70) [35].

The CHRS exhibited acceptable test-retest reliability (ICC = 0.78, 95% CI = 0.63–0.88), which was lower than that reported for the original English version (*r* = 0.96) assessed using a sample in which the majority (67%) of clinical cases involved compulsive hoarding or OCD. We could not preclude the possibility that the general population might harbor a greater potential for changes in buying and cluttering behavior during a 2-week interval, given the compulsive traits among people with hoarding behavior and OCD [36]. Furthermore, our study assessed test-retest reliability using the ICC (model 3) that is a more stringent and recommended method [39], while the original development study adopted the Pearson correlations, which might contribute to an overestimation of the correlation coefficients [45]. Hence, the developers have recently re-examined the test-retest reliability of HRS-I by 11 randomly selected samples with ICC of 0.81–85 [42]. Considering the 2-week test-retest reliability studies of Japanese HRS self-report version (ICC = 0.71 [43];), the current result was comparable and satisfactory.

According to a unified concept of validity that “integrates consideration of content, criteria, and consequences into a construct framework for empirically testing rational hypothesis about score meaning and theoretically relevant relationships” ([31], p. 741), the current results have provided evidence on some aspects of validity of CHRS. The comprehensibility/interpretability and relevance of CHRS was evaluated with satisfactory results, which provided evidence of CHRS items that are relevant to the specification of the boundaries of the construct domain to be assessed [31]. Our factor analysis, which was performed using an EFA and subsequent CFA, supported the unidimensional structure of the CHRS. The one-factor structure of the CHRS demonstrated that all items were satisfactorily clustered into the same domain, with item loadings ranging from 0.69 to 0.85. As the CHRS explained a satisfactory level of the total variance (65.84%), comparable to previous studies [43], we inferred that the one-factor structure of this instrument indicated that compulsive hoarding could be measured consistently using the underlying notion of the DSM-5. It was noteworthy that two pairs of items’ error terms were covaried (i.e., item 2 ‘level of cluttering’ and item 3 ‘difficulties of discarding’, item 2 ‘level of cluttering’ and item 4 ‘excessive acquisition’). These items seem the antecedent’s causes of compulsivity, like a positive feedback mechanism of keeping high input but no output. The remaining two items (item 5 and 6) described the primary consequences of hoarding behaviors (i.e., the emotional distress and functional impairment) [42]. Error terms (representing measurement error of the items) is a unique variance that do not help in the measurement of the latent factor (compulsive hoarding). Some nonrandom measurement errors can be justified reasons for the above error terms being correlated. First, the assessment method of using self-administered survey can be one of nonrandom measurement errors [5]. The HRS originally requires the use of interview as data collection. In order to facilitate the population-based screening of the prevalence of compulsive hoarding, the data collection method of the HRS is converted to self-administered and validated in current study. It is plausible that such change contributes to some common response biases related to self-reporting of the cluttering, discarding and acquisition. In addition, the similarity of sociodemographic background of respondents, like sufficient supply of goods in a well-developed society, can also overestimate the hoarding behavior (i.e., nonrandom errors of measurement in cluttering, discarding and acquisition) of a common non-compulsive hoarder who does not appear any emotional distress and functional impairment.

Unlike the published validation studies of HRS in other countries [11, 29, 43, 44], one merit of the present study is the sequential use of an EFA and CFA to establish the factor structure of the CHRS [24, 27, 35], with a large sample size (820 participants) to fulfil the statistical requirements for these models (EFA, *n* = 300; CFA, *n* = 520) [10, 40]. These results provided evidence of structural aspect of validity that appraises the fidelity of the scoring structure of CHRS to the structure of the construct domain of CHB in Chinese population [31, 35]. However, the current study did not reveal the external aspect of construct validity (i.e., convergent and discriminant evidence, and criterion relevance), and consequential aspect of construct validity (i.e., intended and unintended value implications of test interpretation and use) [31]. Although this study added knowledge to the measurement of CHB, future research should warrant a comprehensive evaluation of remaining aspects of construct validity because validity is an evolving property and validation is an ongoing process [31].

Our study also had some limitations of note. First, the generalizability is limited, as there no people with known hoarding disorder were included in this study and we lacked information about this population. Second, although this is important to validate a self-administered scale for the feasibility of future large-scale population study on screening compulsive hoarding, we could not preclude response bias using self-administered survey. Third, due to insufficient funding, there is no psychiatrist recruited to perform the diagnosis of compulsive hoarding (i.e., served as gold standard) for participants (*N* = 820). Thus, the current research was unable to validate the cut-off value of CHRS through diagnosis accuracy testing. Future study deserves to supplement this missing piece. Fourth, although this study recruited large-scale participants to conduct the validation of CHRS in order to represent general public in Chinese population, the distribution of age of participants was uneven and only 4.4% of them was older than 60. It is noted that samples of older people were insufficient to represent in this study.

## Conclusions

The adaptation of a validated instrument can help to establish an international foundation of scientific knowledge regarding CHB. Our results demonstrate that the CHRS is a contextual relevant, reliable, and valid measure for assessing CHB. The CHRS can be used to assess CHB in the Chinese population in both clinical and research settings. This instrument can assist with the early identification of those at risk of hoarding disorder and the development of appropriate interventions.

## Supplementary information

**Additional file 1.** Chinese Version of Hoarding Rating Scale (CHRS)- Self-administered.

**Additional file 2.** Hoarding Rating Scale (HRS)- Self-administered.

## Data Availability

The (anonymized) datasets analyzed during the current study are available from the corresponding author on reasonable request.

## Abbreviations

CBB: Compulsive hoarding behavior
CFA: Confirmatory factor analysis
CFI: Comparative fit index
CHRS: Chinese Hoarding Rating Scale
CI: Confidence Interval
CVI: Content Validity Index
DSM-5: Diagnostic and Statistical Manual of Mental Disorders, Fifth Edition
HRS: Hoarding Rating Scale
HRS-I: Hoarding Rating Scale-Interview
ICC: Intraclass Correlation Coefficient
I-CVI: Item-level Content Validity Index
IFI: Incremental Fit Index
KMO: Kaiser–Myer–Olkin
NFI: Normed Fit Index
OCD: Obsessive-Compulsive Disorder
RMSEA: Root Mean Square Error of Approximation
S-CVI: Scale-level Content Validity Index
Y-BOCS: Yale-Brown Obsessive Compulsive Scale
χ^2^/d.f.: Chi-square/degree of freedom ratio

## References

[1] American Psychiatric Association (2013). Diagnostic and statistical manual of mental disorders.

[2] Ayers CR, Iqbal Y, Strickland K (2014). Medical conditions in geriatric hoarding disorder patients. Aging Ment Health.

[3] Bollen KA, Stine RA (1992). Bootstrapping goodness-of-fit measures in structural equation models. Soc Methods Res.

[4] Brislin RW, Lonner WJ, Berry JW (1986). The wording and translation of research instrument. Field Methods in Cross-Cultural Research.

[5] Brown TA (2015). Confirmatory factor analysis for applied research.

[6] Byrne BM (2009). Structural equation modeling with AMOS basic concepts, applications, and programming.

[7] Carey EA, de Bolger ADP, Wootton BM (2019). Psychometric properties of the hoarding disorder-dimensional scale. J Obsessive Compuls Relat Disord.

[8] Cath DC, Nizar K, Boomsma D, Mathews CA (2017). Age-specific prevalence of hoarding and obsessive compulsive disorder: a population-based study. Am J Geriatr Psychiatr.

[9] Chen KY, Wang ZH (2010). Advanced statistical analysis using SPSS and AMOS.

[10] Costello AB, Osborne JW (2005). Best practices in exploratory factor analysis: four recommendations for getting the most from your analysis. Pract Assess Res Eval.

[11] Faraci P, Perdighe C, Del Monte C, Saliani AM (2019). Hoarding rating scale-interview: reliability and construct validity in a nonclinical sample. Int J Psychol Psychol Ther.

[12] Farndon J (2010). The World's greatest idea: the fifty greatest ideas that have changed humanity.

[13] Floyd FJ, Widaman KF (1995). Factor analysis in the development and refinement of clinical assessment instruments. Psychol Assess.

[14] Foa EB, Huppert JD, Leiberg S, Langner R, Kichic R, Hajcak G, Salkovskis PM (2002). The obsessive-compulsive inventory: development and validation of a short version. Psychol Assess.

[15] Fontenelle LF, Mendlowicz MV, Soares ID, Versiani M (2004). Patients with obsessive-compulsive disorder and hoarding symptoms: a distinctive clinical subtype?. Compr Psychiatry.

[16] Frost RO, Steketee G, Williams LF, Warren R (2000). Mood: personality disorder symptoms and disability in obsessive compulsive hoarders: a comparison with clinical and monclinical controls. Behav Res Ther.

[17] Frost RO, Tolin DF, Steketee G, Fitch KE, Selbo-Bruns A (2009). Excessive acquisition in hoarding. J Anxiety Disord.

[18] Gaskin J (2012). Confirmatory factor analysis.

[19] Giraudeau B, Mary JY (2001). Planning a reproducibility study: how many subjects and how many replicates per subject for an expected width of the 95 per cent confidence interval of the intraclass correlation coefficient. Stat Med.

[20] Goodman WK, Price LH, Rasmussen SA, Mazure C, Fleischmann RL, Hill CL, Heninger GR, Charney DS (1989). The Yale-Brown obsessive compulsive scale: development, use, and reliability. Arch Gen Psychiatry.

[21] Grisham JR, Frost RO, Steketee G, Kim H, Hood S (2006). Age of onset of compulsive hoarding. J Anxiety Disord.

[22] Hair J, Black W, Babin BYA, Anderson R, Tatham R (2010). Multivariate data analysis: a global perspective.

[23] Jones E (1987). Translation of quantitative measures for use in cross-cultural research. Nurs Res.

[24] Lam C. Development and validation of a quality of life instrument for older Chinese people in residential care homes (doctoral dissertation, the Chinese University of Hong Kong (Hong Kong)): ProQuest dissertations publishing; 2015. 10297281.

[25] Lam SC (2018). Sensitivity and specificity of face validation in determining the comprehensibility of older people on quality of life items. J Am Geriatr Soc.

[26] Lam SC, Chan ZSL, Chong ACY, Wong WWC, Ye J (2018). Adaptation and validation of Richmond compulsive buying scale in Chinese population. J Behav Addict.

[27] Lam SC, Chong ACY, Chung JYS, Lam MY, Chan LM, Shum CY (2020). Methodological study on the evaluation of face mask use scale among public adult: cross-language and psychometric testing. Korean J Adult Nurs.

[28] Lam SC, Yeung CCY, Chan JHM, Lam DWC, Lam AHY, Annesi-Maesano I, Bousquet J (2017). Adaptation of the score for allergic rhinitis in the Chinese population: psychometric properties and diagnostic accuracy. Int Arch Allergy Immunol.

[29] Levy HC, Stevens MC, Tolin DF (2019). Validation of a behavioral measure of acquiring and discarding in hoarding disorder. J Psychopathol Behav Assess.

[30] Li Y, Marques L, Hinton DE, Wang Y, Xiao ZP (2009). Symptom dimensions in Chinese patients with obsessive-compulsive disorder. CNS Neurosci Ther.

[31] Messick S (1995). Validity of psychological assessment: validation of inferences from persons' responses and performances as scientific inquiry into score meaning. Am Psychol.

[32] Morris SH, Jaffee SR, Goodwin GP, Frankln ME (2016). Hoarding in children and adolescents: a review. Child Psychiatry Hum Dev.

[33] Mueller A, Mitchell JE, Crosby RD, Glaesmer H, de Zwaan M (2009). The prevalence of compulsive hoarding and its association with compulsive buying in a German population-based sample. Behav Res Ther.

[34] Polit DF, Beck CT (2006). The content validity index: are you sure you know what's being reported? Critique and recommendations. Res Nurs Health.

[35] Portney LG, Watkins MP (2009). Foundations of clinical research: applications to practice.

[36] Rasmussen JL, Brown TA, Steketee GS, Barlow DH (2013). Impulsivity in hoarding. J Obsess Compulsive Relat Disord.

[37] Samuels JF, Bienvenus OJ, Grados MA, Cullen B, Riddle MA, Liang KY, Eaton WW, Nestadt G (2008). Prevalence and correlates of hoarding behavior in a community-based sample. Behav Res Ther.

[38] Streiner DL (2003). Starting at the beginning: an introduction to coefficient alpha and internal consistency. J Pers Assess.

[39] Streiner DL, Norman GR, Cairney J (2015). Health measurement scales: a practical guide to their development and use.

[40] Tabachnick BG, Fidell LS (2007). Using multivariate statistics.

[41] Tolin DF, Frost RO, Steketee G (2010). A brief interview for assessing compulsive hoarding: the hoarding rating scale-interview. Psychiatry Res.

[42] Tolin DF, Gilliam CM, Davis E, Springer K, Levy HC, Frost RO (2018). Psychometric properties of the hoarding rating scale-interview. J Obsessive Compulsive Relat Disord.

[43] Tsuchiyagaito A, Horiuchi S, Igarashi T, Kawanori Y, Hirano Y, Yabe H, Nakagawa A (2017). Factor structure, reliability, and validity of the Japanese version of the hoarding rating scale-self-report (hrs-sr-J). Neuropsychiatr Dis Treat.

[44] Turna J, Patterson B, Simpson W, Pullia K, Khalesi Z, Kaplan G, Van Ameringen M (2018). Prevalence of hoarding behaviours and excessive acquisition in users of online classified advertisements. Psychiatry Res.

[45] Yen M, Lo LH (2002). Examining test-retest reliability: an intra-class correlation approach. Nurs Res.

