# Supplementary material for: Adaptation and psychometric testing of the hoarding rating scale (HRS): a self-administered screening scale for epidemiological study in Chinese population
Source: BMC Psychiatry. 2020 Apr 14;20:159. doi: 10.1186/s12888-020-02539-7 (PMC7155259; doi:10.1186/s12888-020-02539-7)
Supplement: Supplementary file 1 — Additional file 1. Chinese Version of Hoarding Rating Scale (CHRS)- Self-administered. [file 12888_2020_2539_MOESM1_ESM.doc]

**中文版儲物習慣量表－自我評估**

**Chinese Version of Hoarding Rating Scale (CHRS)- Self-administered**

| **儲物習慣** |
| --- |
| 1. 請問在家中您約有多少件以下物品？    1. 鞋（波鞋、皮鞋等等）__________________對    2. 袋（手袋、背嚢等等）__________________個    3. T恤__________________件    4. 除上述物品外，如適用，在家中您有最多數量的物品是 i. ___＿＿_______，約有 ii. ________件 |

就第2至6題，請您跟據以下準則，**圈**出符合您實際情況的程度。

| 0 沒有困難、困擾或問題  2 少許困難或困擾 / 偶爾 (每周少於1次) 購買不需要的東西 / 購買少量不需要的東西  4 一般困難或困擾 / 恆常 (每周約1至2次) 購買不需要的東西 / 購買一些不需要的東西  6 非常困難或困擾 / 經常 (每周約3至6次) 購買不需要的東西 / 購買很多不需要的東西  8 極度困難或困擾 / 每日購買不需要的東西 / 購買大量不需要的東西 |
| --- |

|  | 沒有　　　少許　　　一般　　　非常　　　極度  困難　　　困難　　　困難　　　困難　　　困難 |
| --- | --- |
| 1. 雜物或大量物品令你使用家居空間上有多大困難？ | ０　 １　 ２　 ３　 ４　 ５　 ６　 ７　 ８ |
| 1. 棄置（或回收／出售／轉贈）一般人會丟棄的物品時，你會有多大程度的困難？ | ０　 １　 ２　 ３　 ４　 ５　 ６　 ７　 ８ |
|  |  |
|  | 沒有　　　少許　　　中等　　　嚴重　　　極大  問題　　　程度　　　程度　　　程度　　　程度 |
| 1. 對於收集贈品、購買多於你需要的東西、或購買負擔不起的東西，這會為你現時帶來多大程度的問題？ | ０　 １　 ２　 ３　 ４　 ５　 ６　 ７　 ８ |
|  |  |
|  | 沒有　　　少許　　　一般　　　非常　　　極度  困擾　　　困擾　　　困擾　　　困擾　　　困擾 |
| 1. 對於雜物、棄置物品、或購買／添置東西的問題，這會為你的情緒帶來多大程度的困擾？ | ０　 １　 ２　 ３　 ４　 ５　 ６　 ７　 ８ |
| 1. 對於雜物、棄置物品、或購買／添置東西的問題，這會為你的生活帶來多大程度的障礙（日常事務、工作／學業、社交活動、家庭活動、經濟困難）？ | ０　 １　 ２　 ３　 ４　 ５　 ６　 ７　 ８ |

Source: Tolin, Frost and Steketee (2010), Psychiatry Research for HRS-I

© David F. Tolin & Simon C. Lam: reproduced with permission of the copyright owners for CHRS- Self-administered.
