# Supplementary material for: Adaptation and psychometric testing of the hoarding rating scale (HRS): a self-administered screening scale for epidemiological study in Chinese population
Source: BMC Psychiatry. 2020 Apr 14;20:159. doi: 10.1186/s12888-020-02539-7 (PMC7155259; doi:10.1186/s12888-020-02539-7)
Supplement: Supplementary file 2 — Additional file 2. Hoarding Rating Scale (HRS)- Self-administered. [file 12888_2020_2539_MOESM2_ESM.doc]

**Hoarding Rating Scale (HRS)- Self-administered**

| **Condition of stocking** |
| --- |
| 1. How many following things do you have at home?    1. __________________ pair/pairs of shoes (Sneakers, leather shoes, etc.)    2. __________________ bag/bags (Satchels, backpacks, etc.)    3. __________________ T-shirt(s)    4. Except the above things, if applicable, the thing with the highest quantity at your home is  (i) ________________，the number of it is (ii) ___________. |

From 2 to 6, please use the following scale when answering items below:

| 0 No problem  2 Mild problem, occasionally (less than weekly) acquires items not needed  4 Moderate, regularly (once or twice weekly) acquires items not needed  6 Severe, frequently (several times per week) acquires items not needed  8 Extreme, very often (daily) acquires items not needed |
| --- |

|  | |
| --- | --- |
|  | | Not at all difficult | Mild | Moderate | Severe | Extremely difficult | | --- | --- | --- | --- | --- | |
| 1. Because of the clutter or number of possessions, how difficult is it for you to use the rooms in your home? | ０ １ ２ ３ ４ ５ ６ ７ ８ |
| 1. To what extent do you have difficulty discarding (or recycling, selling, giving away) ordinary things that other people would get rid of? | ０ １ ２ ３ ４ ５ ６ ７ ８ |

|  | | No problem | Mild | Moderate | Severe | Extreme | | --- | --- | --- | --- | --- | |
| --- | --- | --- | --- | --- | --- | --- |
| 1. To what extent do you currently have a problem with collecting free things or buying more things than you need or can use or can afford? | ０ １ ２ ３ ４ ５ ６ ７ ８ |

|  | | None/not at all | Mild | Moderate | Severe | Extreme | | --- | --- | --- | --- | --- | |
| --- | --- | --- | --- | --- | --- | --- |
| 1. To what extent do you experience emotional distress because of clutter, difficulty discarding or problems with buying or acquiring things? | ０ １ ２ ３ ４ ５ ６ ７ ８ |
| 1. To what extent do you experience impairment in your life (daily routine, job/school, social activities, family activities and financial difficulties) because of clutter, difficulty discarding or problems with buying or acquiring things? | ０ １ ２ ３ ４ ５ ６ ７ ８ |

Source: Tolin, Frost and Steketee (2010), Psychiatry Research for HRS-I

© David F. Tolin & Simon C. Lam: reproduced with permission of the copyright owners for HRS- Self-administered.
